# Liver metastatic recurrence after curative endoscopic submucosal dissection for slightly submucosal invasive gastric cancer: A case report and literature review

**DOI:** 10.1002/deo2.70041

**Published:** 2025-02-07

**Authors:** Masahiro Niikawa, Teppei Akimoto, Kumiko Kirita, Yuji Yoshida, Tomomi Okubo, Korenobu Hayama, Tsutomu Hatori, Osamu Goto, Shunji Fujimori, Katsuhiko Iwakiri

**Affiliations:** ^1^ Department of Gastroenterology Nippon Medical School Chiba Hokusoh Hospital Chiba Japan; ^2^ Department of Gastroenterology Nippon Medical School Hospital Tokyo Japan; ^3^ Department of Pathology Nippon Medical School Chiba Hokusoh Hospital Chiba Japan

**Keywords:** eCuraB, endoscopic submucosal dissection, gastric cancer, metastasis, recurrence

## Abstract

In Japan, differentiated‐type early gastric cancer with submucosal invasion <500 µm, tumor diameter <3 cm, no lymphovascular invasion, and negative resection margin are included in pathological curative criteria after endoscopic resection (ER). However, there are a few reports presenting local or metastatic recurrence after ER for the lesions described above. A 72‐year‐old man was diagnosed with early gastric cancer and underwent endoscopic submucosal dissection (ESD). Pathological diagnosis was well‐differentiated tubular adenocarcinoma, 8 × 6 mm, T1b1(SM1; 428 µm below the muscularis mucosae), negative lymphovascular invasion, and negative resection margin, which was included in curative criteria. Eighteen months after ESD, the laboratory studies indicated an increase in carcinoembryonic antigen of 17.6 ng/mL (normal range <5 ng/mL). While endoscopy showed no local recurrence finding, contrast‐enhanced computed tomography (CT) showed a metastatic liver tumor in S4. Gadolinium ethoxybenzyl diethylenetriamine pentaacetic acid‐enhanced magnetic resonance imaging and ^18^F‐fluorodeoxyglucose positron emission tomography/CT revealed metastatic liver tumors in S4, S5, and S8. The liver biopsy specimen showed tubular adenocarcinoma and the findings of immunochemical staining were similar to that of the specimen of prior ESD. Thus, he was diagnosed with multiple liver metastatic recurrences after curative ER. Currently, it has been 3 years and 5 months since ESD and 1 year and 11 months since liver metastatic recurrence, and the patient has survived receiving 5th‐line systemic chemotherapy. Here, we report a rare case of liver metastatic recurrence 18 months after curative ESD for early gastric cancer.

## INTRODUCTION

Endoscopic submucosal dissection (ESD) is widely accepted as one of the curative endoscopic treatments for early gastric cancer (EGC). Although the initial absolute indication for endoscopic resection (ER) was defined as differentiated‐type adenocarcinoma (≤2 cm in diameter), the indications have been gradually expanded based on the results of clinical trials. In Japan, the pathological diagnosis of differentiated‐type adenocarcinoma (≤3 cm in diameter), pT1b1(SM1; <500 µm below the muscularis mucosa) with negative margins and negative lymphovascular invasion is classified as endoscopic curability B (eCuraB), which is included in curative resection and follow‐up without additional treatments is recommended.^1^ However, there are a few reports that EGCs classified as eCuraB developed local or metastatic recurrence during surveillance after ER. We experienced a case of multiple liver metastatic recurrences after curative ER for pT1b(SM1) gastric cancer which was classified as eCuraB.

## CASE REPORT

**FIGURE 1:**
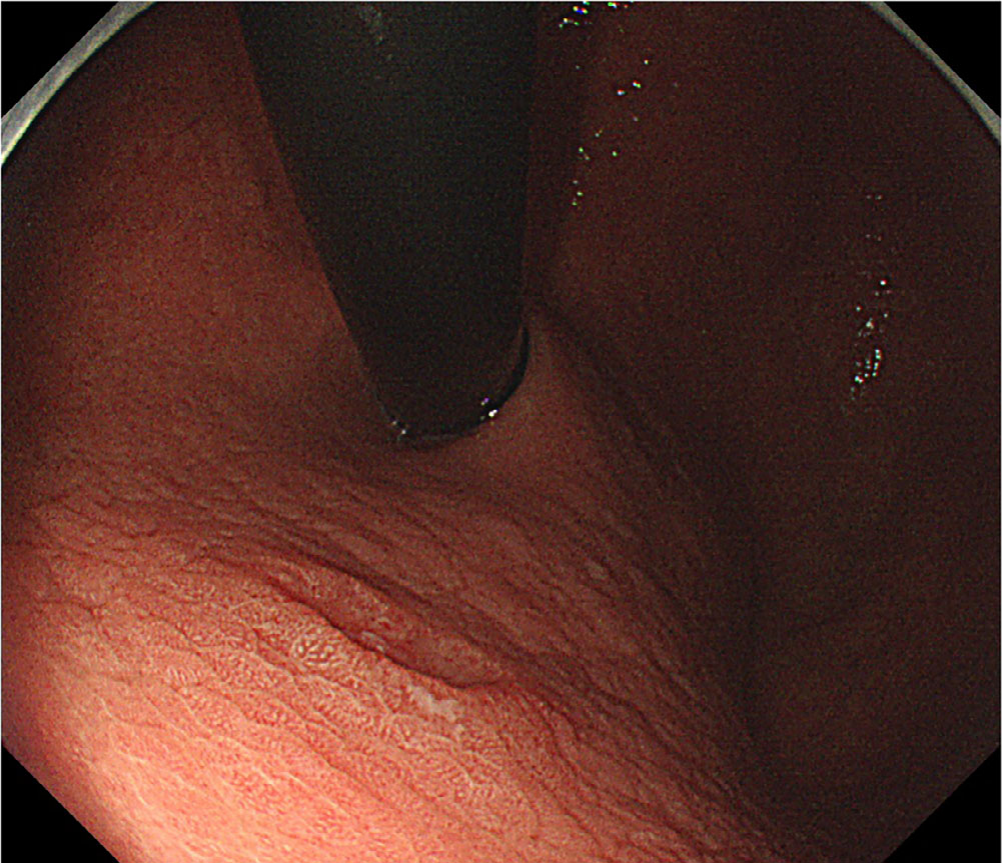
Esophagogastroduodenoscopy showing early gastric cancer on the lesser curvature of the upper gastric body.

**FIGURE 2:**
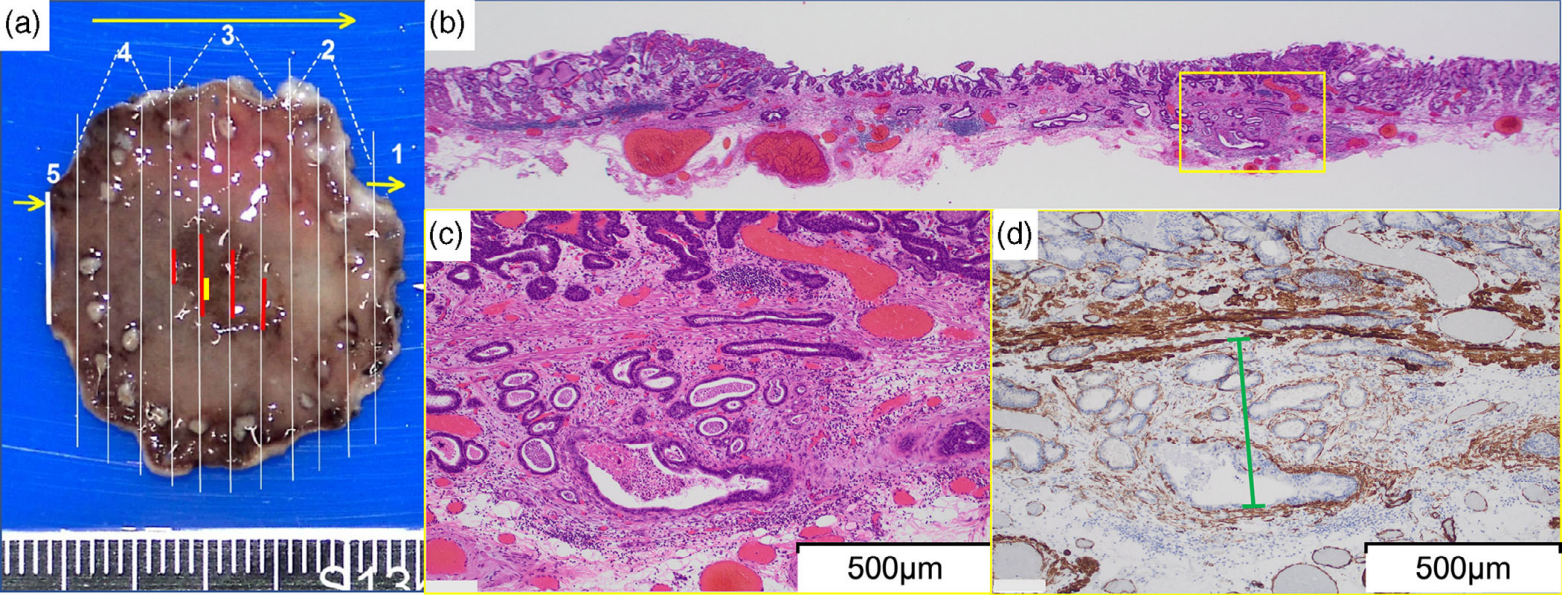
Pathological findings. (a) The endoscopic submucosal dissection specimen was made by 2 mm width. The red lines indicate the cancer and the yellow line indicates the submucosal invasion part. (b) Hematoxylin and eosin staining of a section showing submucosal invasion which is indicated by the yellow square. Pathological examination revealed a type 0–IIc lesion without ulceration, 8 × 6 mm in size, which has invaded the submucosa with negative lymphovascular involvement and tumor‐free margins. (c) A magnified image of the submucosal invasion part indicated by the yellow square. (d) Immunohistochemical evaluation of desmin staining of submucosal invasion part. The submucosal invasion distance indicated by the green line is 428 µm.

A 72‐year‐old man was referred to our hospital for treatment of EGC detected by annual esophagogastroduodenoscopy (EGD). There was no medical history of other malignant tumors. *Helicobacter pylori* was eradicated 1 year before the referral. The lesion was preoperatively diagnosed as a type 0–IIc, intramucosal well‐differentiated adenocarcinoma, 8 mm in size on the lesser curvature of the upper gastric body (Figure 1). While considering the potential risk of submucosal invasion based on the distinct depressed finding of the lesion, we screened metastatic lesions. Serum tumor markers (carcinoembryonic antigen (CEA) and carbohydrate antigen 19‐9 (CA19‐9)) were within normal range and contrast‐enhanced computed tomography (CT) showed no findings of lymph node or distant metastasis. The clinical diagnosis was stage IA gastric adenocarcinoma (cT1aN0M0) and ESD was performed as a curative treatment. The lesion was resected with en bloc resection (Figure 2a). Pathological findings were well‐differentiated tubular adenocarcinoma, 8 × 6 mm, T1b1(SM1; 428 µm below the muscularis mucosae), no ulceration, no lymphovascular invasion, and negative resection margins (Figure 2b–d). The pathological sections were prepared at 2 mm intervals. Lymphovascular invasion was evaluated by immunostaining with D2‐40, Factor VIII, and Elastica‐van Gieson staining, and was confirmed negative.

**FIGURE 3:**
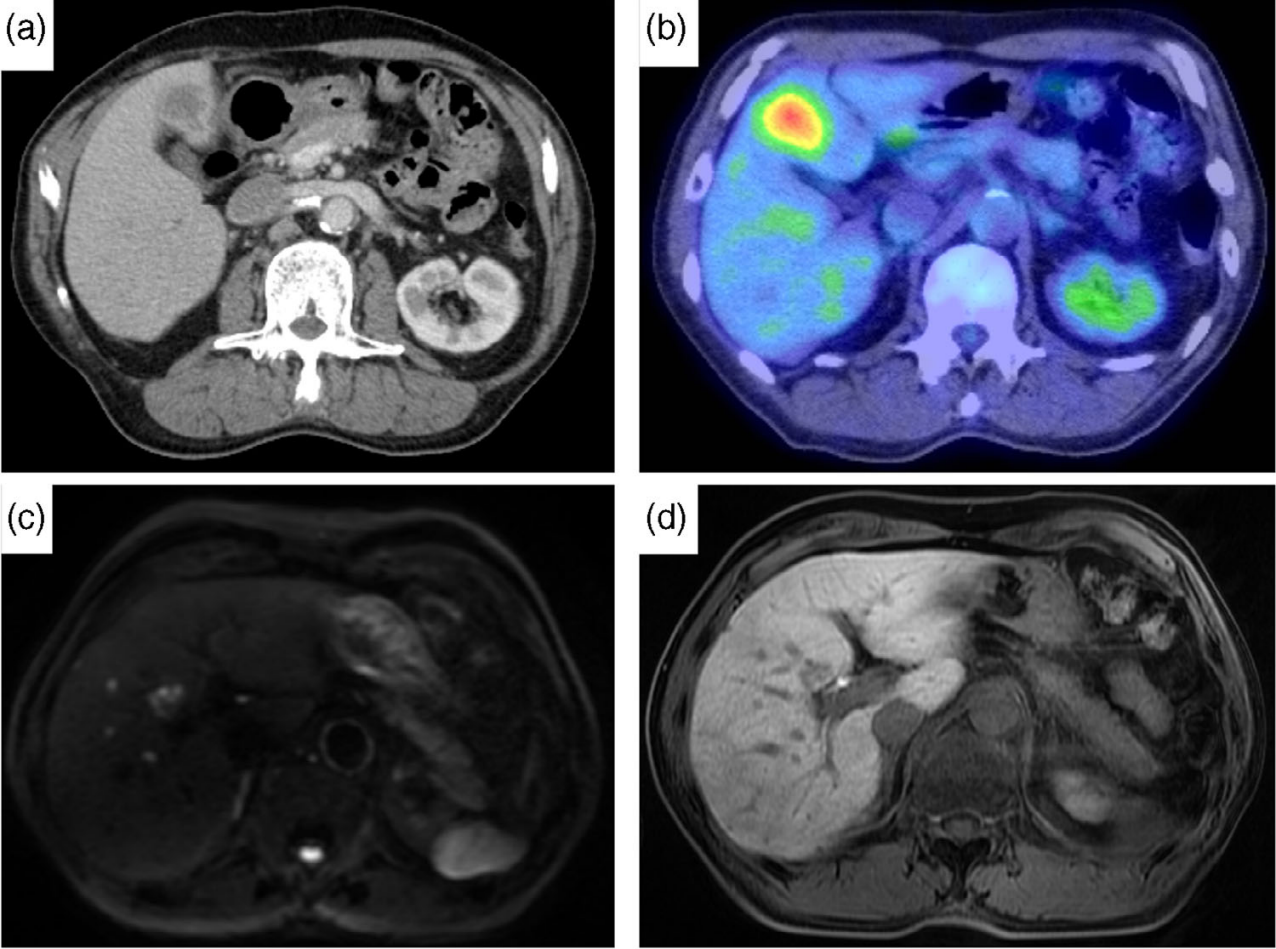
Computed tomography (CT), gadolinium ethoxybenzyl diethylenetriamine pentaacetic acid (Gd‐EOB‐DTPA)‐enhanced magnetic resonance imaging (EOB‐MRI), and 18^F^‐fluorodeoxyglucose positron emission tomography (FDG‐PET)/CT images 18 months after curative endoscopic submucosal dissection. (a) CT revealed a metastatic liver tumor in S4. (b) FDG‐PET/CT showing FDG uptake in the liver. (c, d) EOB‐MRI revealed metastatic liver tumors in S4, S5, and S8.

According to the Japanese Gastric Cancer Treatment Guideline 2021 (6th edition), curative resection (eCuraB) was achieved in the present case.^1^ He was followed up by biannual CT and laboratory studies including serum tumor markers (CEA and cCA19‐9) and annual EGD, which indicated no findings of local and metastatic recurrence within 1 year. However, 18 months after ESD, the laboratory studies indicated an increase in CEA of 17.6 ng/mL (normal range, <5 ng/mL), and CA19‐9 was within the normal range. EGD did not show local recurrence findings, on the other hand, Abdominal CT detected a metastatic liver tumor in S4 (Figure 3a). ^18^F‐fluorodeoxyglucose (FDG) positron emission tomography/CT showed FDG uptake in the same sites of the liver (Figure 3b). Gadolinium ethoxybenzyl diethylenetriamine pentaacetic acid (Gd‐EOB‐DTPA) enhanced magnetic resonance imaging revealed metastatic liver tumors in S4, S5, and S8 (Figure 3c,d). We performed a liver biopsy to confirm the histological findings of multiple liver tumors. The pathological finding of the liver biopsy specimen was tubular adenocarcinoma and immunochemical staining was similar to that of the resected ESD specimen (negative expression of cytokeratin‐7, cytokeratin‐20, MUC5AC, MUC6, alpha‐fetoprotein, SALL4, and Glypican‐3). Thus, we diagnosed liver metastasis recurrence after curative ESD. Currently, it has been 3 years and 5 months since ESD and 1 year and 11 months since recurrence, and the patient is receiving 5th‐line systemic chemotherapy with controlled multiple liver metastasis recurrence.

## DISCUSSION

According to the current 6th edition of the Guidelines for Gastric Cancer Treatment, pathological curative criteria of ER for EGC consist of two categories; eCuraA and eCuraB.^1^ eCuraA includes an intramucosal differentiated‐type cancer (size not limited), intramucosal differentiated‐type cancer with ulcerative findings (≤3 cm in diameter), or undifferentiated‐type cancer (≤2 cm in diameter) with negative margins and negative lymphovascular invasion.^1^ Then, eCuraB is defined to fulfill the following pathological findings: differentiated type‐dominant ≤3 cm in diameter, pT1b1(SM1; <500 µm from the muscularis mucosae), negative margins, and negative lymphovascular invasion.^1^ However, if the undifferentiated component is included in the portion of submucosal invasion, the lesions are excluded from the curative criteria.^1^ In the previous guideline, undifferentiated‐type cancer described above is classified as eCuraB, but the current guideline has included it in eCuraA based on the prospective clinical study.^1^, ^2^, ^3^ Thus, only differentiated type‐dominant ≤3 cm in diameter, pT1b1(SM1) cancer is classified as eCuraB in the current guideline.^1^

Follow‐up strategies differ for eCuraA and eCuraB resection because there is a difference between the evidence of long‐term clinical studies for eCuraA and those for eCuraB; therefore, eCuraB requires surveillance of metastases.^1^ Annual EGD is recommended for eCuraA resection and annual or biannual EGD and abdominal ultrasonography or CT are recommended for eCuraB resection. In the present case, the lesion was classified as eCuraB and the patient underwent annual EGD, biannual CT, and laboratory studies including tumor markers (CEA and CA19‐9). Subsequently, 18 months after ESD, multiple liver metastases were observed. A multicenter prospective cohort study of long‐term survival after ER of EGC showed that the proportions of local recurrence and metastatic recurrence after eCuraB resection were 0% (0/387) and 0.52% (2/387), respectively.^4^ Although infrequent, there is the potential risk of local or metastatic recurrence after eCuraB resection. To the best of our knowledge, including our case, there have been seven reported cases (searched in PubMed and Ichushi‐Web (NPO Japan Medical Abstracts Society) databases) of local recurrence, lymph node metastasis, or distant metastases after ER for pT1b1(SM1) cancer classified as eCuraB (Table 1).^5^, ^6^, ^7^, ^8^, S2,S3

**TABLE 1:** Characteristics of patients with recurrences after curative endoscopic submucosal dissection for minute submucosal invasive gastric cancer.

Case	Author/year	Age/Sex	Location	Circumference	Shape	Size (mm)	Differentiation	Depth	Ulcerative findings	Recurrence	Period until recurrence (month)
1	Takii M /2012	52/female	Middle	Lesser curvature	0‐IIa+IIc	13	Well‐Differentiated Tubular Adenocarcinoma	SM1	‐	Local Lymph node	4
2	Oya H/2012	65/male	Lower	Anterior wall	0‐IIc	21	Well‐Differentiated Tubular Adenocarcinoma	SM1 (<500 µm)	+	Lymph node	48
3	Tanabe S/2014	73/male	No data	No data	0‐IIc	13	Well‐Differentiated Tubular Adenocarcinoma	SM1	‐	Lymph node Liver Pleura	55
4	Abe S/2015	66/male	Middle	Lesser curvature	0‐IIa	16	Well‐Differentiated Tubular Adenocarcinoma	SM1 (100µm)	+	Local Lymph node Adrenal gland Peritoneal dissemination	86
5	Byung‐Hoon M/2015	66/male	Lower	Lesser curvature	0‐IIc	12	Moderately Tubular Adenocarcinoma	SM1	‐	Lymph node	49
6	Hagiwara K/2022	81/male	Upper	Posterior wall	0‐IIb	30	Well‐Differentiated Tubular Adenocarcinoma	SM1 (480 µm)	‐	Local Lymph node	27
7	Our case/2023	74/male	Upper	Posterior wall	0‐IIc	8	Well‐Differentiated Tubular Adenocarcinoma	SM1 (428 µm)	‐	Liver	18

Endoscopic characteristics showed that the most common macroscopic type was a superficial depressed type (71.4%, 5/7 cases), and location and circumference were varied. The most common metastatic type was lymph node metastasis (85.7%, 6/7 cases), and all except for one case had recurred within 5 years. All cases of recurrence after eCuraB showed lymph node metastasis and the diameters of the lesions were 12 mm or more except for our case. The diameter of our case was 8 mm and distant metastasis was observed without lymph node metastasis, which is a characteristic finding not seen in previous cases. This suggests that even small tumor size is at risk for distant metastasis after eCuraB.

Based on the Japanese Classification of Gastric Carcinoma (15th edition), for the pathological evaluation after ESD, fixed specimens were sectioned at intervals of approximately 2 mm and evaluated in all sections.^S1^ Sako et al. reported that immunostaining with D2‐40 is useful for the diagnosis of lymphatic invasion because of the difficulty of detecting lymphatic vessels with conventional hematoxylin and eosin staining.^9^ In our case, lymphovascular invasion was evaluated by immunostaining with D2‐40, Factor VIII, and Elastica‐van Gieson staining, and was confirmed negative. Kumei et al. also reported that since it is an evaluation of step sections and not a comprehensive evaluation of the entire lesion, there is the existence of potential lymphovascular invasion, massive submucosal invasion, or positive resection margin in the un‐sectioned specimens, and additional sections would change pathological curative criteria of ER.^10^ The risk of these pathological evaluations is also always present after not only eCuraB but also eCuraA.

In summary, we report a rare case of distant metastatic recurrence after curative ER for pT1b(SM1) gastric cancer. It should be noted that recurrence after eCuraB resection is rare, but surveillance for metastatic recurrence with abdominal ultrasonography or CT and laboratory studies including tumor makers is recommended in addition to EGD.

## CONFLICT OF INTEREST STATEMENT

None.

## Supporting information



S1. Japanese Gastric Cancer Assosiation. Japanese classification of gastric carcinoma: 15th edition. Kanehara Shuppan 2017 (in Japanese)
