# Supplementary material for: Liver metastatic recurrence after curative endoscopic submucosal dissection for slightly submucosal invasive gastric cancer: A case report and literature review
Source: DEN Open. 2025 Feb 7;5(1):e70041. doi: 10.1002/deo2.70041 (PMC11805676; doi:10.1002/deo2.70041)
Supplement: Supplementary file 1 — S1. Japanese Gastric Cancer Assosiation. Japanese classification of gastric carcinoma: 15th edition. Kanehara Shuppan 2017 (in Japanese) [file DEO2-5-e70041-s001.docx]

**Supporting information**

Supplemental references

S1. Japanese Gastric Cancer Assosiation. Japanese classification of gastric carcinoma: 15th edition. *Kanehara Shuppan* 2017 (**in Japanese**).

S2. Hagiwara K, Nishimaki H, Yamashita H. A case of early gastric cancer that developed local recurrence and nodal metastases after eCura B ESD. *Endoscopia Digestiva* 2022; **34**: 1189-95 (**in Japanese**).

S3. Tanabe S, Ishido K, Matsumoto T *et al.* Clinicopathological features, progress, and prognosis in cases of local recurrence and metastatic recurrence following endoscopic submucosal dissection for gastric cancer : A multi-institutional joint study. *Stomach and Intestine* 2014; **49**: 1601-8 (**in Japanese**).
